# Atypical respiratory distress in eastern Democratic Republic of the Congo prior to the COVID-19 pandemic. A case report

**DOI:** 10.1186/s40794-021-00134-6

**Published:** 2021-04-06

**Authors:** Guy-Quesney Mateso, Marius Baguma, Pacifique Mwene-Batu, Ghislain Maheshe Balemba, Fabrice Nzabara, Samuel Makali, Aline Bedha, Bonheur Furaha, Jimmy Minani, Christian Tshongo Muhindo, Espoir Bwenge Malembaka, Mannix Imani Masimango, Tony Akilimali Shindano, Justin Cirhuza Cikomola, Kanigula Mubagwa

**Affiliations:** 1 Department of Internal Medicine, Hôpital Provincial Général de Référence de Bukavu (HPGRB), Bukavu, Democratic Republic of the Congo; 2 Faculty of Medicine, Université Catholique de Bukavu (UCB), Bukavu, Democratic Republic of the Congo; 3 École Régionale de Santé Publique (ERSP), Université Catholique de Bukavu (UCB), Bukavu, Democratic Republic of the Congo

**Keywords:** COVID-19, SARS-Cov-2 coronavirus, Pandemic, Democratic Republic of the Congo, DRC, Case report

## Abstract

**Background:**

Predictions have been made that Africa would be the most vulnerable continent to the novel Coronavirus disease 2019 (COVID-19). Interestingly, the spread of the disease in Africa seems to have been delayed and initially slower than in many parts of the world. Here we report on two cases of respiratory distress in our region before the official declaration of the disease in December 2019, cases which in the present times would be suspect of COVID-19.

**Case presentation:**

These two cases (one 55-year-old man and one 25-year-old woman) of acute respiratory distress secondary to atypical pneumonia were seen in Bukavu, in Eastern Democratic Republic of the Congo (DRC), between September and December 2019. One patient had returned from China and the other had close contacts with travellers from China in the 2 weeks prior to the onset of symptoms. In either case, the aetiology could not be accurately determined. However, the two cases presented a clinical picture (progressive dyspnoea, preceded by dry cough and fever) and laboratory changes (procalcitonin within the normal range, slight inflammation, and lymphopenia) compatible with a viral infection. The chest X-ray series of the first patient showed lesions (reticulations, ground glass, and nodules ≤6 mm) similar to those currently found in COVID-19 patients. In addition, unlike the 25-year-old female patient who had no comorbidity, the 55-year-old male patient who had hypertension as comorbidity, developed a more severe acute respiratory distress which progressed to death.

**Conclusion:**

These cases bring to the attention the fact that COVID-19-like syndromes may have already been present in the region months before the official beginning of the pandemic. This also brings to question whether a prior presence of the disease or infections with related virus may account for the delayed and less extensive development of the pandemic in the region.

## Background

The world is currently facing a devastating pandemic of the novel coronavirus disease 2019 (COVID-19), caused by severe acute respiratory syndrome coronavirus 2 (SARS-CoV-2), which was first declared in Wuhan, China, in December 2019 [1]. Clinically, the infection by the virus is asymptomatic in most cases [2, 3]. In many cases, it may manifest itself by non-typical symptoms such as fever, cough, sore throat [4–8], known to be present in common cold. In some other cases more evocative symptoms such as anosmia and ageusia may be present [9–12]. Most severe cases develop respiratory distress with hypoxia [5, 6]. Laboratory findings include an inflammatory syndrome, mild lymphopenia and an increased level of cytokines [13]. Pneumonia is present on chest X-rays, and more specific aspects such as ground-glass images can be detected on radiography or CT scan [5–8]. Although the first COVID-19 cases were described in China [4–8], the spread of the disease in other countries and regions, such as Western Europe, United States, South America (e.g., Brazil), or South Africa has been higher than in China [14]. As of February 22, 2021, over 111 million cases of COVID-19 have been confirmed in 192 countries, including more than 2.46 million deaths [14, 15]. Predictions have been made that Africa would be the most vulnerable continent to COVID-19, because of various factors: dense population in many cities, close physical contacts indoors due to poor housing conditions or outdoors due to the fact that many people rely on daily income from small business, high prevalence of chronic diseases (e.g., infections by human immunodeficiency virus (HIV), malnutrition, etc.) interfering with the immune defence, absent or poor infrastructures to diagnose or treat patients, and very frequent travels between China and Africa [16, 17]. Sub-Saharan African countries are shown to have the world’s highest estimated vulnerability index to infectious disease outbreaks [18]. The Democratic Republic of the Congo (DRC) is considered to be among the countries at highest risk, especially since travel continued even after the pandemic was declared [16].

Interestingly, except for South Africa, the spread of the disease in the rest of sub-Saharan Africa seems to have been delayed and occurred to a relatively smaller extent than in many parts of the world. Since the first COVID-19 case was officially identified in Africa (in Egypt) on February 14, 2020 [19, 20], 2,780,836 confirmed cases and 70,107 deaths have been documented as of February 21, 2021, about 1 year later [14]. These numbers are far below those recorded in other parts of the world. In the DRC, the first case has been reported on March 10, 2020, in Kinshasa (12 million inhabitants), but as of February 19, 2021, only 19,047 confirmed cases have been registered in the same city and 25,080 countrywide [21]. In Bukavu (about 1 million inhabitants, density: 16,600 inhabitants/km^2^), the capital city of South-Kivu, in Eastern DRC, the first two COVID-19 cases were travellers who arrived on March 18 and 20, 2020, but despite a delay in quarantine measures (started on March 30, 2020), nearly 1 year later the province has registered only 726 confirmed cases of which 56 were deaths [22].

The reasons for the low rate of COVID-19 propagation in Africa remain unknown. Recent data suggest a relatively high level of infection with other coronaviruses [23, 24]. Although it remains unclear whether such infections are associated with cross-immunity and increased resistance to COVID-19, it is of interest to document and test cases of severe respiratory distress presenting with symptoms like those of COVID-19. In this report, we present two patients who were admitted between September and December 2019 for acute respiratory distress in the Intensive Care Unit (ICU) of the “Hôpital Provincial Général de Référence de Bukavu” (HPGRB), in Bukavu. For both patients, a diagnosis of atypical viral pneumonia was made. COVID-19 was not yet known at that time; hence the patients were not tested for the disease, but the clinical presentation, radiological findings, and the course of the disease are similar to those of COVID-19.

Our report is timely in that it coincides with a moment the debate about herd immunity is rife in many countries in Europe and North America which are grappling with successive waves of COVID-19 and societal impacts of containment measures. Documenting the presence of other viral infections on the African continent way longer before the COVID-19 pandemic unveiled, may help understand the seemingly downward trends in the COVID-19 spread on the continent, which contrasts with the severe damages (higher numbers of hospitalisations, intensive care admissions, and deaths) as in Europe and the Americas. Our report also comes to further underpin the urgent call for inclusion of Africa in the current planning and prioritization processes for global COVID-19 research [25].

## Methods

Upon learning of coronavirus, we revisited the files of a few patients who had been hospitalized during the last term of 2019, and enrolled the two patients presented in this report, who had been admitted with fever, cough, and hypoxia with signs of atypical pneumonia on chest radiography.

We reviewed their medical records to collect information on physical examination, and the laboratory (including haematology, biochemistry, and microbiology tests) as well as the chest X-ray findings. Complete blood count was carried out using an automated haematology analyser (Edan H50, Hamburg, Germany). Biochemical tests on plasma or serum were done using semi-automatic analysers (CYANSmart CY009, CYPRESS Diagnostics, Langdorp, Belgium, for creatinine and blood urea nitrogen; Rayto Chemray 120, Shenzhen, China, for alanine transaminase, aspartate aminotransferase, alkaline phosphatase, albumin, and bilirubin; iCHROMA II, Brussels, Belgium, for D-dimer and procalcitonin; EDAN i15 analyser, Hamburg, Germany, for arterial blood gas). Bacteriological tests included blood cultures performed in BACT/ALERT® FA Plus and BACT/ALERT® FN culture media (bioMerieux, Marcy-l’Etoile, France), screening of tuberculosis using a Ziehl-Neelsen stain of sputum, and microscopic examination of Giemsa-stained thick blood film for diagnosis of clinical malaria. At the time of the hospitalization of the two patients, there was no capacity for regular testing for the presence of respiratory virus. HIV test was carried out on serum samples using rapid point-of-care test Determine™ HIV-1/2 Ag/Ab Combo (Alere Inc., Florida, USA) which detects HIV antigens as well as antibodies [26]. Chest X-rays were carried out using Perlong PL 50DR Digital Radiography (Nanjing, China).

No ethical permission was needed since the study involved a retrospective review of data from already treated patients, whose identity was kept confidential.

## Case presentation

### Case 1

**Fig. 1:**
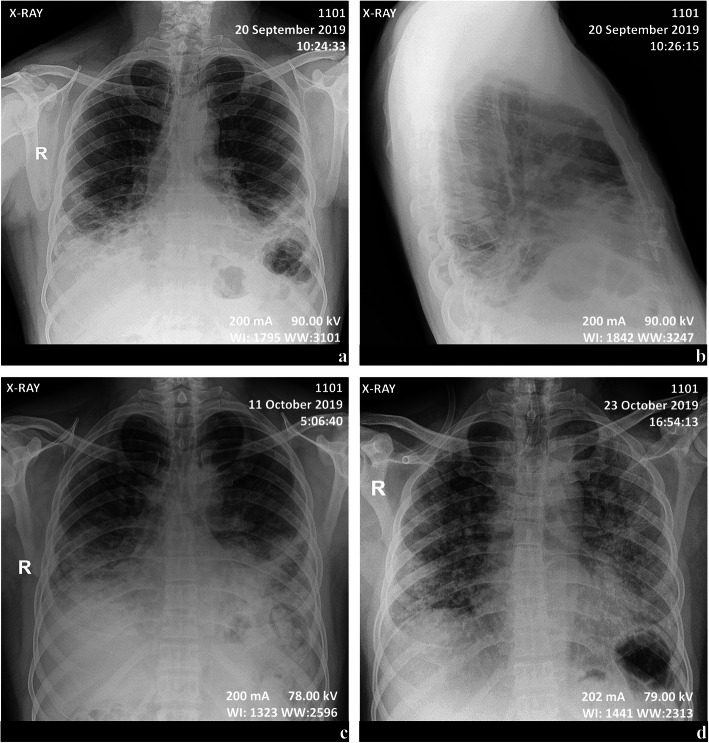
Serial chest X-rays of Patient 1. Multifocal then diffuse airspace disease in a 55-years-old man. Bibasal lung consolidation with assorted trabeculae, especially in the posterior regions, overhung by discreet hazy opacities (**a**, **b**). Follow-up showing a cranially progress of the predominant inhomogeneous consolidation still coexisting with a clearly diffuse ground-glass (**c**). There is apparent replacement of alveolar densification by diffuse reticular interstitial marks with some few parahilar nodules (**d**)

A 55-year-old man, with a 10-year history of hypertension (treated with nicardipine 20 mg twice daily, bisoprolol 5 mg twice daily and aldactazine 1 tablet once daily) and a 5-year history of pigeon breeding, was seen in the Department of Internal Medicine on September 20, 2019, for dry cough, 2 weeks after returning from Guangzhou (Guangdong province, China). The patient did not complain of any loss of smell or taste. He had good mental orientation. Axillary temperature was 37 °C. Chest auscultation noted normal heart sounds and normal vesicular breath sounds without rales or sibilance. There was no abdominal tenderness nor enlarged liver or spleen. There was no low limb oedema. There was no loss of peripheral motor or sensor function and no other abnormal neurological signs. Laboratory results showed normal complete blood count (CBC) and procalcitonin (< 0.1 ng/ml); an elevated serum creatinine (1.8 mg/dl, hence a glomerular filtration rate, GFR, of 50.6 ml/(min*1.73m^2^)) and electrolytes disturbances (low sodium at 130 mmol/l, low potassium at 3.4 mmol/l and low calcium 1.04 mmol/l) (Table 1). A diagnosis of non-specific interstitial pneumonia was evoked based on chest X-rays showing bi-basal and posterior densification (25 to 30% of total chest height), blurring heart borders and diaphragm, associated with trabecular bands and ground-glass zones on the mid third of the lungs (Fig. 1a, b). He was sent back home with oral azithromycin (500 mg daily for 5 days), paracetamol (1000 mg up to 3 times a day if necessary) and an antitussive drug.

**Table 1:** Summary of laboratory tests for the 55-year-old male patient

Laboratory tests	Normal values	Sept. 20	Oct. 10	Oct. 14	Oct. 17	Oct. 23	Oct. 28
Arterial Blood Gas							
pH	7.35–7.45	–	7.55	7.52	7.55	7.31	6.97
PaO_2_ (mmHg)	75–100	–	33	48	49	60	60
PCO_2_ (mmHg)	35–45	–	29.7	36.5	31.6	43	74
SaO_2_ (%)	94–100	–	74	88	89	92	73
Lactates (mmol/L)	< 2.00	–	1.89	4.00	3.27	3.1	12.2
Bicarbonates (mmol/L)	22.0–26.0	–	25.1	28.8	27.0	21	16.5
P_a_O_2_/FiO_2_ (mmHg)	400–500	–	157	229	233	60	60
Complete blood count							
White blood cells (× 10^3^/μL)	4.00–10.00	8.00	11.60	–	–	–	7.60
Neutrophils (×10^3^/μL)	1.50–7.00	5.46	9.07	–	–	–	5.40
Lymphocytes (×10^3^/μL)	1.50–4.50	1.63	1.38	–	–	–	1.43
Monocytes (× 10^3^/μL)	0.20–1.00	0.74	1.11	–	–	–	0.74
Eosinophils (×10^3^/μL)	0.10–0.50	0.16	0.02	–	–	–	0.03
Red blood cells (× 10^6^/μL)	4.20–5.70	5.28	4.90	–	–	–	5.41
Haemoglobin (g/L)	13.0–18.0	16.0	15.3	17.0	–	–	16.5
Haematocrit (%)	40.0–52.0	45.1	42.3	50.0	–	–	48
Platelets (×10^3^/μL)	150–450	244	213	–	–	–	156
Electrolytes							
Sodium (mmol/L)	135–155	130	129	135	132	–	133
Potassium (mmol/L)	3.5–5.1	3.4	3.4	3.2	3.4	–	3.7
Chloride (mmol/L)	96–115	99	99	102	99	–	115
Calcium (mmol/L)	1.10–1.40	1.04	1.12	1.21	1.25	–	1.33
Magnesium (mg/dL)	1.60–2.50	–	–	1.8	–	–	1.7
C-Reactive protein (mg/L)	< 3.0	–	54.9	–	98	43	102
Procalcitonin (ng/mL)	< 0.10	< 0.10	< 0.10	–	–	0.69	< 0.1
Creatinine (mg/dL)	0.6–1.40	1.80	2.04	–	–	1.80	3.8
Blood Urea Nitrogen	8.0–23.0	–	40.0	–	–	107	150
Blood Glucose (mg/dl)	60–110	82	127	–	–	82	–
Hb1Ac (%)	4–5.70	3.29	–	–	–	–	–
Troponin (ng/ml)		0.02	–	–	–	–	–
Pro-BNP (pg/ml)		–	183.4	–	–	101.5	–
D-dimer (μg/L)	< 500	–	790	–	–	–	–
Thick blood film	Negative	–	Negative	–	–	Negative	–
Haemocultures	Negative	–	Negative	–	–	Negative	–
Ziehl-Neelsen stain of sputum	Negative	–	Negative	–	–	Negative	–
Serologies							
HIV	Negative	–	Negative	–	–	–	–
Viral hepatitis B	Negative	–	Negative	–	–	–	–
Viral hepatitis C	Negative	–	Negative	–	–	–	–
Syphilis	Negative	–	Negative	–	–	–	–

Despite treatment, coughing was exacerbated, and fever and dyspnoea occurred 2 weeks later. On October 10, 2019, he was admitted in the Emergency ward. The clinical evaluation noticed polypnea (30 breaths/min), tachycardia (100 beats/min) and fever (38 °C). Oxygen saturation (SaO_2_) measured with a pulse oximeter was 54% [normal range: 95–100%]. The patient presented a respiratory distress (nose flaring, chest retractions and cyanosis) and had fine crackles at both lung bases. Cardiovascular examination was normal. Laboratory results showed a moderate inflammatory syndrome with hyperleukocytosis (11,600 leucocytes/μl [4000-10,000/μl] of which 9071 neutrophils [1500-7000/μl] and 1380 lymphocytes [1500-4500/μl]) and increased C-reactive protein (CRP, 54.9 mg/l [0–3 mg/l]). Procalcitonin levels were normal [below 0.1 ng/ml]. An arterial blood gas (ABG) analysis revealed the presence of severe hypoxemia (P_a_O_2_ 33 mmHg [75–100 mmHg], SaO_2_ 74%) and respiratory alkalosis (pH 7.55 [7.35–7.45], P_a_CO_2_ 29.7 mmHg [35–45 mmHg], P_a_O_2_/FiO_2_ ratio 157 mmHg [400–500 mmHg], bicarbonate 25.1 mmol/l [22–26 mmol/l], and lactates 1.89 mmol/l [< 2 mmol/l]). Bacteriological investigations were all negative. Tuberculosis was unlikely given a Ziehl-Neelsen stain of sputum which was negative for three samples collected at different times. HIV serological tests were negative (Table 1). A second chest X-rays showed persistence of the aforementioned findings, except for the trabeculae hidden by the alveolar densification that had progressed up to the level of the main bronchi, overhung by a well delimited ground glass zone without encroachment upon the apices (Fig. 1c).

The patient was hospitalized in the ICU for acute respiratory distress syndrome secondary to viral pneumonia with probable bacterial superinfection. He received oxygen by mask (5 l/min) and intravenous antibiotics (combination of amoxicillin and clavulanic acid 1/0.25 g thrice daily). Despite this treatment, continuous fever (average temperature: 38.5 °C) and hypoxemia persisted, and lactates increased to 4 mmol/l. So, 2 days later, amoxicillin and clavulanic acid were replaced by levofloxacin (500 mg) and ceftriaxone (1 g), both twice daily, in addition to intravenous dexamethasone (16 mg thrice daily).

Four days later, no improvement was observed. The oxygen flow was increased to 10 l/min and dexamethasone replaced by methylprednisolone (125 mg twice daily for 5 days). During the following week, there was a slight improvement of the dyspnoea and fever, but on October 23, 2019, fever reappeared, and the respiratory distress worsened. A new chest X-ray showed a reduction of the alveolar consolidation to the advantage of an interstitial syndrome (reticulations, ground-glass, and nodules ≤6 mm) extending to lung tops (Fig. 1d). Meanwhile, the CRP was 49 mg/l and procalcitonin 0.62 ng/ml. A diagnosis of respiratory zoonosis was considered, given the history of pigeon breeding. Intravenous methylprednisolone (125 mg twice daily) was reintroduced and Duovent® (combined ipratropium and fenoterol) was administered in nebulization, without any improvement. SaO_2_ (measured with a pulse oximeter) remained at 40%, P_a_O_2_/F_i_O_2_ ratio decreased to less than 70 mmHg, and the patient became confused, with loss of notion of space and time, probably because of the hypoxia.

**Fig. 2:**
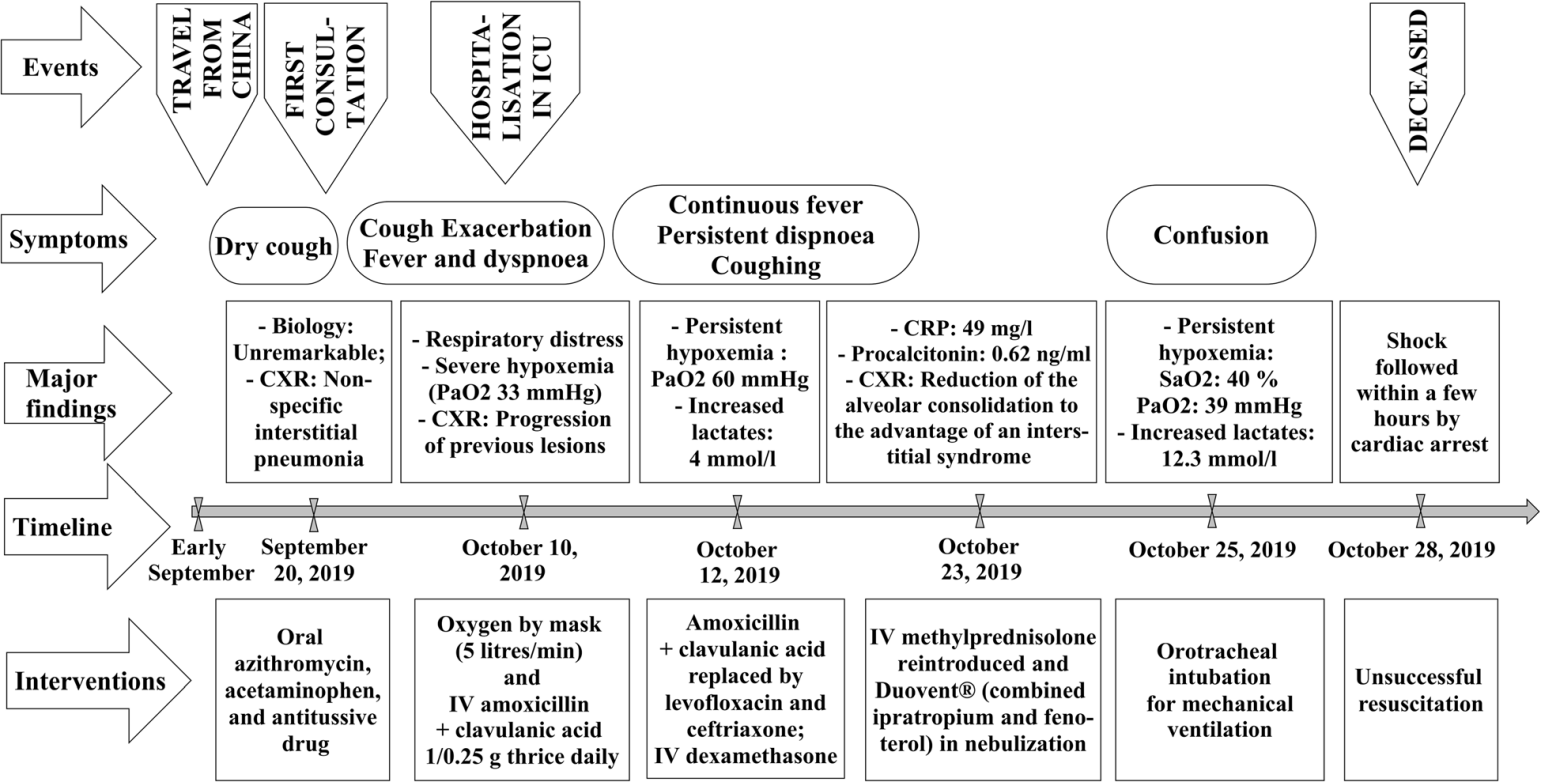
Summary of the 55-year-old patient’s information and care organized as a timeline. CRP: C-reactive protein; CXR: chest X-rays; ICU: Intensive care unit; IV: intravenous; SaO2: Oxygen saturation; PaO2: partial pressure of oxygen in the arterial blood

On October 25, 2019, the patient underwent orotracheal intubation for mechanical ventilation. This improved the SaO_2_ to 90–94% but on October 28, 2019, the patient developed a shock, with increased lactacidemia (12.2 mmol/l), followed within a few hours by cardiac arrest irresponsive to resuscitation (Fig. 2).

### Case 2

A 25-year-old woman, with no history of recent travel, was admitted at the Emergency ward on December 18, 2019, for a one-week progressive dyspnoea, preceded by dry cough and fever. She had no particular medical history and was a nurse in a hospital where Chinese employees from a multinational mining company are treated, of whom some had recently travelled from China (the place they came from in China could not be determined) and three of them had presented flu-like symptoms within 2 weeks. On admission, she could not complete sentences due to dyspnoea. At ambient air, she presented a SaO_2_ of 82% and signs of respiratory distress without cyanosis. Her pulmonary and cardiac auscultation was normal.

**Fig. 3:**
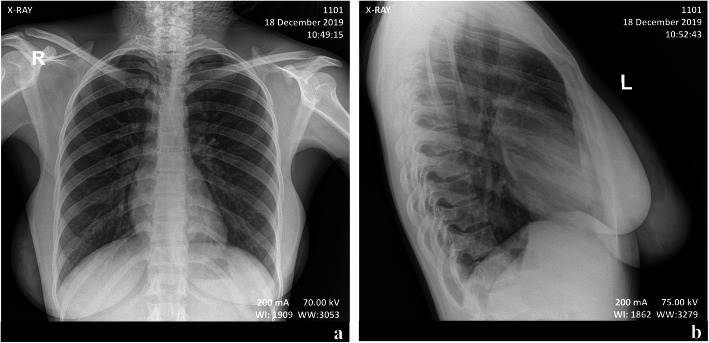
Chest X-rays of Patient 2. Subnormal chest X-rays of a 25-year-old female. Discrete peribronchovascular blur in the posterobasal regions. Normal aspect of the hila and the costophrenic angles

The chest X-rays showed reticular lines and peribronchovascular haziness in the infrahilar and retrocardiac regions, bilaterally. This suggested a mild interstitial pneumonia (Fig. 3a, b). Laboratory results showed a slight inflammation with CRP at 14.5 mg/l, lymphopenia (700 lymphocytes/μl) and normal procalcitonin (< 0.1 ng/ml). The ABG showed a hypoxemia (P_a_O_2_ 60 mmHg) and a respiratory alkalosis (pH 7.51, P_a_CO_2_ 35 mmHg, P_a_O_2_/F_i_O_2_ 286 mmHg, bicarbonate 27.1 mmol/l, and lactates 1.50 mmol/l). Creatinine, blood urea nitrogen and blood electrolytes were normal. HIV serology was negative.

**Fig. 4:**
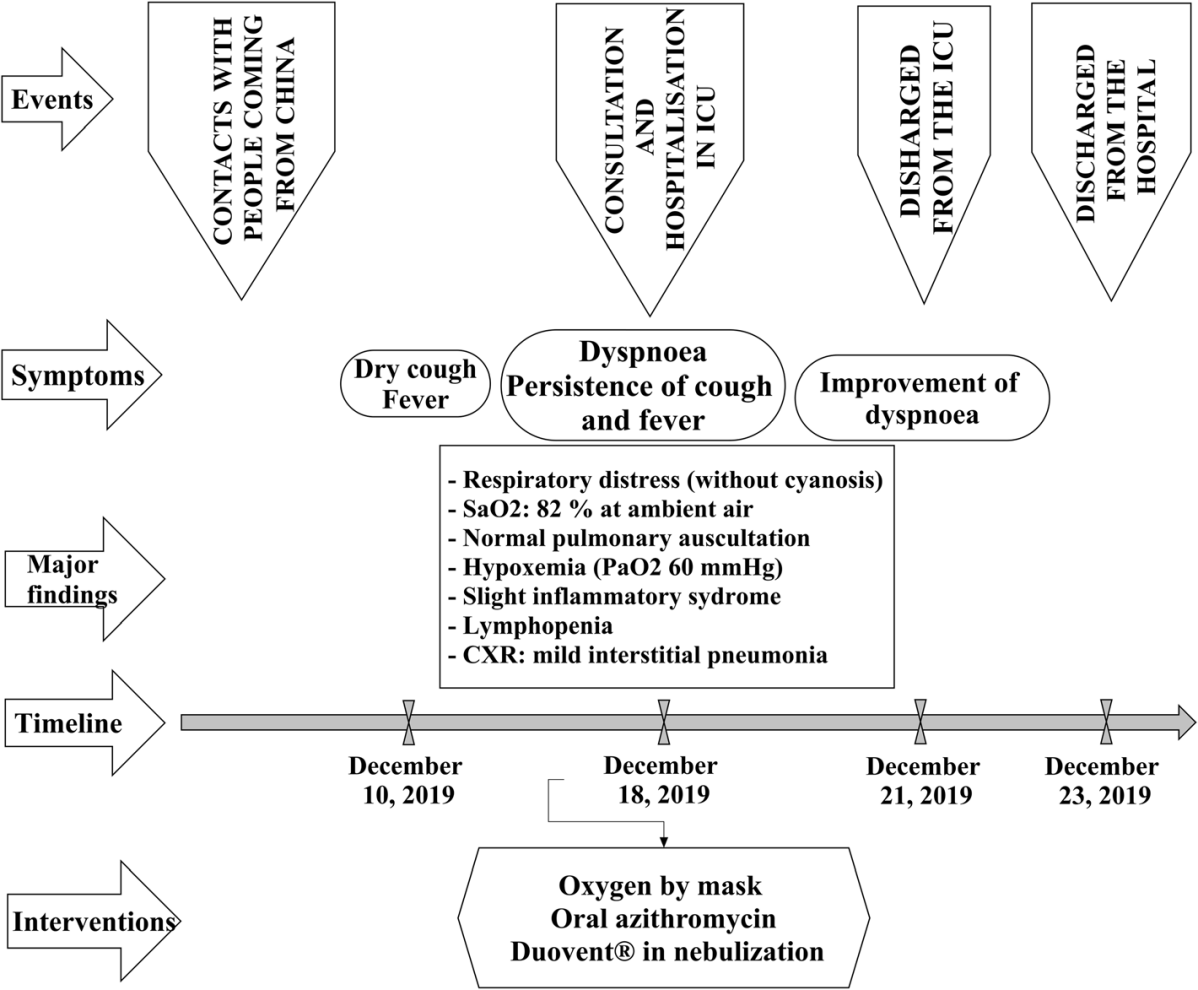
Summary of the 25-year-old patient’s information and care organized as a timeline. CXR: chest X-rays; ICU: Intensive care unit; SaO2: Oxygen saturation; PaO2: partial pressure of oxygen in the arterial blood

A diagnosis of moderate acute respiratory distress syndrome secondary to a viral pneumonia was retained and the patient was admitted in the ICU, receiving oxygen (4 l/min), azithromycin (500 mg once daily for 5 days) and Duovent® in nebulization. Three days later, she was eupnoeic with normal SaO_2_ at ambient air. She was discharged from hospital 5 days after admission (Fig. 4).

## Discussion

In this report, we present two cases of acute respiratory distress secondary to atypical pneumonia in eastern DRC. One patient had returned from China and the other had had close contacts with employees who had returned from a travel to China in the 2 weeks prior to the onset of symptoms. In one case, the respiratory disease did not respond to treatments for typical and atypical causes of respiratory failure. In the second case, hypoxia was disproportionate with the chest lesions. In no case could the aetiology be accurately determined. COVID-19 was not yet known when these patients were hospitalized. However, when viewed retrospectively the two cases presented a clinical picture and laboratory changes (procalcitonin within the normal range, slight inflammation, and lymphopenia) compatible with a viral infection. In both cases, alkalosis was present as also found by others in COVID-19 patients [27]. The chest X-ray series of the first patient showed lesions similar to those found in COVID-19 [28]. In addition, unlike the 25-year-old female patient who had no comorbidity, the 55-year-old male patient who had hypertension as comorbidity, developed a more severe acute respiratory distress which progressed to death. For COVID-19, available data show that older age and comorbidities such as cardiovascular diseases, diabetes, hypertension, chronic respiratory diseases and cancer are associated with an increased risk of death [29, 30].

The other most common cause of acute respiratory distress syndrome due to viral infections is influenza. Previous data by others suggest that ground glass opacities are less common in influenza [31]. The ground glass lesions detected in our patients are indeed more compatible with COVID-19, but it should be recognized that such radiological findings are not pathognomonic and do not allow to exclude non-COVID-19 infections. Compared to COVID-19, influenza is more frequently associated with productive cough and with less gastrointestinal symptoms [32]. Cough was indeed non-productive in our patients, but there was no report of gastro-intestinal symptoms or of loss of taste and/or smell to more strongly support the possibility of COVID-19. A diagnosis of psittacosis can hardly be sustained in view of the dramatic evolution to death despite the use of antibiotics, including a macrolide and a quinolone, which are known to be efficacious against Chlamydophila psittaci [33]. In retrospect, especially given the context of travel to China or of contact with travellers from China, the question can thus be raised as to whether these cases could have been COVID-19 infections.

The two cases presented here are illustrative of (unpublished) observations made by clinicians in Bukavu during the last term of 2019. During that period, an abnormally high number of patients presented to local hospitals with symptoms of cough and fever, which were diagnosed as flu or atypical pneumonia for those who could have a chest radiography.

The COVID-19 epidemic has apparently been late to hit Africa, relative to the starting dates in Asia or Europe. This is not due to lack of contacts with China, known to be the origin of the pandemic. During the past two decades business exchanges between many regions of Africa, especially the eastern part of the continent, with Asia, and in particular with China, have been on the increase [34–36]. Even after the official declaration of the pandemic, many persons from Africa continued to travel to China, to secure the import of various goods. The apparent delay in the outbreak of COVID-19 cannot therefore be explained solely on the grounds that there is a low level of traveling between Africa and China [17, 36].

In addition, despite the evidence of community spread of COVID-19 in DRC since nearly 1 year, the progression of the number of such cases has been relatively slower compared to Europe, the USA, and Latin America. One reason for the small number of reported positive cases could arguably be due to the low level of testing. However, indirect evidence of the local propagation of the disease consists in the occurrence of an increased number of people consulting for cough, fever, dyspnoea and eventually for severe respiratory distress requiring respiratory assistance as it has been the case from the last week of May 2020 up to mid-July 2020 [22]. From mid-March 2020 when the first COVID-19 were reported in South-Kivu up to end-May 2020, such an increased incidence of cases of acute respiratory distress was not observed locally. The question is to which extent potential mechanisms underlying the relative protection involve factors such as the generally young age of the population, other immunizations (Bacille Calmette-Guérin vaccination, measles, etc.), some environmental or genetic factors, etc.

In view of the above-mentioned observations, it would also be tempting to raise the question as to whether SARS-CoV-2 infection might have already been present in the region prior to the officially declared start in China in December 2019 and account for the fact that the disease did not develop into an epidemic for so many months. Reports of COVID-19 cases before the declaration of the pandemic exist [37]. It is important to stress the limitations of using retrospective case reports (especially limited to only 2 patients) as evidence of suspected COVID-19, especially in contexts such as ours with limited detailed information on travel/contact history and with limited conservation of blood or other samples for retrospective laboratory analysis.

## Conclusion

This case report highlights the fact that clinical cases presenting like COVID-19 were present in our part of eastern DRC months before the official start of the pandemic. This raises the question as to whether prior infection with SARS-Cov2 or by other infections causing cross-immunity could explain the late and smaller extent of the COVID-19 pandemic in the region. We wish to carry out larger studies, including retrospective microbiological and serological investigations in local communities in order to test this hypothesis. There is also a need to develop more robust epidemiological surveillance systems in Africa, sensitive to the threat of pandemics as a consequence of increased international traffic and globalisation.

## Data Availability

All data generated or analysed during this study are included within the article.

## Abbreviations

ABG: Arterial blood gas
COVID-19: The novel coronavirus disease 2019
CRP: C-reactive protein
CXR: Chest X-Rays
DRC: The Democratic Republic of the Congo
HIV: Human Immunodeficiency Virus
HPGRB: Hôpital Provincial Général de Référence de Bukavu
ICU: Intensive Care Unit
PaO_2_: Partial pressure of oxygen in the arterial blood
SaO_2_: Oxygen saturation
SARS-CoV-2: Severe acute respiratory syndrome coronavirus 2
